# 2657. Investigation of *Respiratory Syncytial Virus (RSV)* Stability in Nasal Aspirate Collected from *RSV* Infected Patients

**DOI:** 10.1093/ofid/ofad500.2268

**Published:** 2023-11-27

**Authors:** Atsuko Yamamoto, Yoko Kajiwara, Takamichi Baba, Saori Okaga, Mayumi Kakui, Takao Shishido

**Affiliations:** SHIONOGI & CO., LTD., Toyonaka, Osaka, Japan; SHIONOGI & CO., LTD., Toyonaka, Osaka, Japan; Shionogi & Co., Ltd, Toyonaka, Osaka, Japan; Shionogi TechnoAdvance Research & Co., Ltd., Toyonaka, Osaka, Japan; Shionogi TechnoAdvance Research & Co., Ltd., Toyonaka, Osaka, Japan; SHIONOGI & CO., LTD., Toyonaka, Osaka, Japan

## Abstract

**Background:**

RSV is the leading cause of serious acute lower respiratory infection in infants and various therapeutic agents are under investigation. For reliable study of antiviral effect of the agent in clinical study/trial, appropriate specimen storage which keeps virus stability is needed. WHO recommends specimens to be stored in transport medium and placed at 4°C. In case the specimens cannot be processed within 48 hours, they are recommended to be frozen at or below -70 °C. However, there is no investigation of RSV stability of specimen stored under these conditions. In this study, we investigated the RSV stability of nasal aspirates stored in universal transport medium (UTM) at 4°C or frozen by evaluating infectious virus titer.

**Methods:**

Infectious virus titer was measured by immunostaining HEp-2 cells infected with RSV. To assess the variability of virus titration assay data, aliquots of RSV propagated with HEp-2 cells (RSV stocks) were titrated in three independent experiments and standard deviation (SD) was calculated. When the variation of virus titer from baseline was within 3SD, RSV was considered to be stable. As a preliminary experiment, virus stability of RSV stock stored in UTM at 4°C was evaluated. The impact of freezing method on RSV stability was also assessed. Nasal aspirates from 16 patients with confirmed RSV infection by rapid diagnostic test were collected and stored in UTM at 4°C up to 9 days with titration at 2 or 3 timepoints. Duration of stable RSV was estimated by statistical analysis of virus titer with regression model. RSV stability of specimen stored at -80°C for 10-15 months was also assessed.

**Results:**

SD of RSV stock titration data was 0.3 log_10_TCID_50_/mL. RSV was considered to be stable when the variation of virus titer from baseline was within 1.0 log_10_TCID_50_/mL. Virus of RSV stock stored in UTM at 4°C was stable up to 120 hours. Sample freezing in deep freezer affected RSV stability, whereas freezing in liquid nitrogen or dry ice-ethanol bath kept RSV stability. RSV in nasal aspirates collected and stored in UTM at 4°C was estimated to be stable for 2 days. RSV in the specimen stored at -80°C for 10-15 months was stable.

**Conclusion:**

RSV in nasal aspirate collected and stored in UTM at 4°C was estimated to be stable for 2 days. RSV in specimen stored at -80°C suggested to be stable for 1 year and more.

**Disclosures:**

**All Authors**: No reported disclosures

